# Ineffective Absorption? Failure of Direct-Acting Therapy for Chronic Hepatitis C in Cirrhotic Patients With Roux-en-Y Gastric Bypass

**DOI:** 10.1177/2324709619858127

**Published:** 2019-06-19

**Authors:** Clara Y. Tow, John F. Reinus

**Affiliations:** 1 Montefiore Medical Center, Bronx, NY, USA

**Keywords:** hepatitis C virus, direct-acting antiviral, cirrhosis, Roux-en-Y, gastric bypass

## Abstract

In this era of direct-acting antiviral (DAA) therapy for chronic hepatitis C virus (HCV) infection, treated patients have extremely high rates of sustained virologic response to short courses of therapy regardless of stage of fibrosis. Treatment failure is uncommon and often attributed to medication noncompliance or viral resistance to drug. This report describes 2 Child-Pugh-A cirrhotic patients who failed to clear HCV in response to therapy with DAAs. Each patient had Roux-en-Y gastric bypass (RYGB) surgery preceding DAA therapy. RYGB may create multiple barriers to adequate DAA absorption as a result of changes in gastrointestinal physiology. Treatment monitoring and duration should be carefully considered in this unique patient population.

## Introduction

Direct-acting antiviral (DAA) drugs have increased the rate of sustained virologic response (SVR) to therapy of chronic hepatitis C virus (HCV) infection in treatment-naive, genotype-1 cirrhotic patients from 52% after 48 weeks of combination treatment with interferon and ribavirin to over 90% after 12 weeks of combination treatment with sofosbuvir and simeprevir.^1^ Multiple DAAs have since become available and achieved even higher rates of SVR across all subgroups of treatment-naive and treatment-exposed HCV-infected patients.^2,3^ Treatment failures, therefore, are uncommon and often caused by medication noncompliance or less commonly by viral drug-resistance mutations.^4,5^

Patients who undergo Roux-en-Y gastric bypass (RYGB) have a small gastric pouch often less than 10% of the original volume of the stomach.^6^ The pouch is anastomosed to the jejunum, thus bypassing the duodenum and dissociating bile salts from digestible contents. This surgery causes early satiety and malabsorption to promote weight loss. These physiological alterations can further impact drug absorption, though altered pharmacokinetics have been poorly described.^7^

This report describes 2 chronic HCV patients with compensated cirrhosis with RYGB anatomy who did not achieve SVR with DAA therapy.

## Case Descriptions

### Case 1

A 63-year-old man with chronic genotype-1A HCV infection complicated by compensated cirrhosis (Child-Turcotte-Pugh Score A [CTP-A], Model for End-Stage Liver Disease [MELD] 6) with radiographic and laboratory evidence of portal hypertension was evaluated for HCV treatment. He previously had been treated with multiple courses of interferon and ribavirin with end-of-treatment responses but subsequent relapses. He also had been treated with interferon, ribavirin, and a protease inhibitor, but discontinued the medications prematurely due to adverse drug effects. The patient had RYGB surgery in early 2000.

In 2014, he was treated with a combination of sofosbuvir and simeprevir. Prior to therapy, his liver enzyme levels were normal and his HCV RNA level was 29 964 IU/mL. After the first 4 weeks of treatment, HCV RNA was undetectable but it became detectable again by the 16th week of treatment. In 2015, he was treated with sofosbuvir and ledipasvir for 24 weeks. He had no detectable HCV RNA 12 weeks after completion of treatment (SVR-12) but relapsed 24 weeks later.

### Case 2

A 57-year-old woman with chronic genotype-1A HCV infection complicated by cirrhosis (CTP-A, MELD 6) with a history of grade 1 hepatic encephalopathy was evaluated for HCV treatment. She previously had been treated unsuccessfully with interferon and ribavirin. She had RYGB surgery in the 1990s.

In 2014, she had elevated liver enzyme levels (aspartate aminotransferase = 88 U/L, alanine aminotransferase = 76 U/L) and an HCV RNA level of 4 136 276 IU/mL. She was treated with a combination of sofosbuvir and simeprevir for 12 weeks. HCV RNA level during treatment is unknown; however, 8 weeks after completing therapy, her HCV RNA level was 3 132 997 IU/mL. Subsequently, she was treated with a combination sofosbuvir and ledipasvir, but this therapy was stopped after 11 weeks due to lack of virologic response.

## Discussion

HCV DAA treatment failures are uncommon in patients with compensated cirrhosis. Many viral resistance substitutions and polymorphisms have been described, but these mutations do not necessarily predict treatment failure, particularly in patients receiving second-generation DAAs.^8^ While 14% to 18% of patients in the ION-1 and ION-2 studies had evidence of NS5A resistance prior to treatment with sofosbuvir and ledipasvir, SVR rates in treated patients remained high, even in treatment-experienced cirrhotic patients (89% with NS5A resistance vs 96% without resistance).^2,9^

In this case series, both patients had recognized predictors of poor response to DAA therapy: genotype-1a virus, prior exposure to a protease inhibitor in 1 patient, and exposure to first-generation DAAs in both patients.^10,11^ Given these patients’ altered gastrointestinal anatomy, we propose that altered drug delivery leading to inadequate serum levels may also have contributed to treatment failure.

An acidic environment is required for optimal absorption of some DAAs.^12^ Ledipasvir is insoluble at a pH > 7.5. After RYGB surgery, patients have decreased gastric acid production because the majority of acid-producing parietal cells are located in the body of the stomach, which has been separated surgically from the remnant pouch. Physiologic studies have confirmed that acid production in a RYGB pouch is significantly less than that in matched controls with normal gastrointestinal anatomy.^13^ Sometimes, RYGB can promote bile acid reflux into the gastric pouch due to lack of a pyloric sphincter, further raising the gastric pH.^14^ Finally, the site of DAA drug absorption has not been established with certainty.^15^ As the majority of the stomach and proximal small intestine are no longer in linear continuity with the proximal digestive tract after RYGB, it is possible that after RYGB, DAAs lack access to absorption sites.

Digestive anatomy and physiology are significantly altered by RYGB, creating multiple potential barriers to adequate DAA absorption and increasing the risk of DAA treatment failure. While the specific cause or causes of DAA failure in these 2 cases are unknown, our experience in treating these patients draws attention to the fact that a different approach to HCV therapy may be necessary in patients with altered gastrointestinal anatomy. Consideration should be given to terminate hepatitis C treatment in patients who do not have early viral suppression in the first month of therapy in order to prevent prolonged, futile drug exposure that may foster development of DAA resistance. Drugs potentially less susceptible to resistance mutations and alterations in absorption, such as glecaprevir/pibrentasvir, which does not contain an NS5A inhibitor, should be chosen to treat patients with hepatitis C and abnormal gastrointestinal anatomy.

## References

[1] AASLD/IDSA HCV Guidance Panel. Hepatitis C guidance: AASLD-IDSA recommendations for testing, managing, and treating adults infected with hepatitis C virus. Hepatology. 2015;62:932-954. 26111063 10.1002/hep.27950

[2] Afdhal N Reddy KR Nelson DR et al Ledipasvir and sofosbuvir for previously treated HCV genotype 1 infection. N Engl J Med. 2014;370:1483-1493. 24725238 10.1056/NEJMoa1316366

[3] Wilson EM Kattakuzhy S Sidharthan S et al Successful retreatment of chronic HCV genotype-1 infection with ledipasvir and sofosbuvir after initial short course therapy with direct-acting antiviral regimens. Clin Infect Dis. 2016;62:280-288. 26521268 10.1093/cid/civ874 PMC4706633

[4] Benitez-Gutiérrez L Barreiro P Labarga P et al Prevention and management of treatment failure to new oral hepatitis C drugs. Expert Opin Pharmacother. 2016;17:1215-1223. 27149603 10.1080/14656566.2016.1182156

[5] Colpitts CC Baumert TF. Addressing the challenges of hepatitis C virus resistance and treatment failure. Viruses. 2016;8:E226. 27537906 10.3390/v8080226 PMC4997588

[6] Abdeen G le Roux CW Mechanism underlying the weight loss and complications of Roux-en-Y gastric bypass. Review. Obes Surg. 2016;26:410-421. 26530712 10.1007/s11695-015-1945-7 PMC4709370

[7] Hachon L Declèves X Faucher P Carette C Lloret-Linares C. RYGB and drug disposition: how to do better? Analysis of pharmacokinetic studies and recommendations for clinical practice. Obes Surg. 2017;27:1076-1090. 28124236 10.1007/s11695-016-2535-z

[8] Jacobson IM. The HCV treatment revolution continues: resistance considerations, pangenotypic efficacy, and advances in challenging populations. Gastroenterol Hepatol (N Y). 2016;12(10 suppl 4):1-11. PMC5210027 28070174

[9] Afdhal N Zeuzem S Kwo P et al Ledipasvir and sofosbuvir for untreated HCV genotype 1 infection. N Engl J Med. 2014;370:1889-1898. 24725239 10.1056/NEJMoa1402454

[10] Arias A Aguilera A Soriano V et al Rate and predictors of treatment failure to all-oral HCV regimens outside clinical trials. Antivir Ther. 2017;22:307-312. 27341294 10.3851/IMP3061

[11] Akuta N Sezaki H Suzuki F et al Retreatment efficacy and predictors of ledipasvir plus sofosbuvir to HCV genotype 1 in Japan. J Med Virol. 2017;89:284-290. 27357737 10.1002/jmv.24617

[12] German P Mathias A Brainard D Kearney BP. Clinical pharmacokinetics and pharmacodynamics of ledipasvir/sofosbuvir, a fixed-dose combination tablet for the treatment of hepatitis C. Clin Pharmacokinet. 2016;55:1337-1351. 27193156 10.1007/s40262-016-0397-0

[13] Smith CD Herkes SB Behrns KE Fairbanks VF Kelly KA Sarr MG. Gastric acid secretion and vitamin B12 absorption after vertical Roux-en-Y gastric bypass for morbid obesity. Ann Surg. 1993;218:91-96. 8328834 10.1097/00000658-199307000-00014 PMC1242905

[14] Swartz DE Mobley E Felix EL. Bile reflux after Roux-en-Y gastric bypass: an unrecognized cause of postoperative pain. Surg Obes Relat Dis. 2009;5:27-30. 19095503 10.1016/j.soard.2008.10.009

[15] de Kanter CT Drenth JP Arends JE et al Viral hepatitis C therapy: pharmacokinetic and pharmacodynamic considerations. Clin Pharmacokinet. 2014;53:409-427. 24723109 10.1007/s40262-014-0142-5

